# Increased dietary iron alters taxonomic composition and function of zebrafish gut microbiome

**DOI:** 10.17912/micropub.biology.001220

**Published:** 2026-04-14

**Authors:** Stuart Gordon, Sam Evans, Kobie Kirven, Megan Whisonant

**Affiliations:** 1 Biology, Presbyterian College, Clinton, SC, US; 2 Biology Department, Presbyterian College, Clinton, SC, US

## Abstract

Gut microbiota are crucial to both gastrointestinal tract health and host well-being. Oral iron supplementation is commonly used, but knowledge of iron’s impact on the gut microbiome is limited. Using Zebrafish ( *Danio rerio* ) as a model organism, we tested effects of increased dietary iron on gut taxonomic composition and function. Increased dietary iron significantly altered the zebrafish microbiome taxonomic composition and enriched physiological conditions of aerobic respiration. Mass spectrometry (GCMS and LCMS), utilized to measure primary metabolite and lipid levels, pointed to significant increases in amino acids under increased iron supplementation, but no significant change in lipid metabolite levels.

**Figure 1. Effects of increased dietary iron on zebrafish gut microbiome composition and functional output:** Figure 1. Increased dietary iron was found to alter the taxonomic composition of bacteria present in the gut and the metabolic composition of the gut. Control and experimental groups received a standard 309 ppm of dietary iron and an increased 366 ppm of dietary iron respectively and are illustrated within each panel. Figure 1 **A)** Bar plot of bacterial composition at the phylum level of both control and experimental groups reflecting the relative frequency of each class. **B)** Interleaved box and whisker plots of high frequency classes found in both control and experimental groups. Significant differences in relative frequencies between control (blue bars) and experimental (red bars) are illustrated for Gammaproteobacteria and Alphaproteobacteria frequencies, but no significant difference was found for Clostridia. **C)** Principal component analysis (PCoA) Plot of Jaccard dissimilarity representing beta diversity Control variables as blue points (n=5), and experimental variables as red points (n=5, points are overlapping on the plot). **D)** Heatmap of primary metabolite concentrations for control and treatment samples. The first three columns depict controls while the last three columns depict the treatment groups. Significant increases for several amino acids were discovered. **E)** PCoA plot showing overlap of concentrations in lipids between control and experimental samples. Control variables are red points and experimental variables are green points.

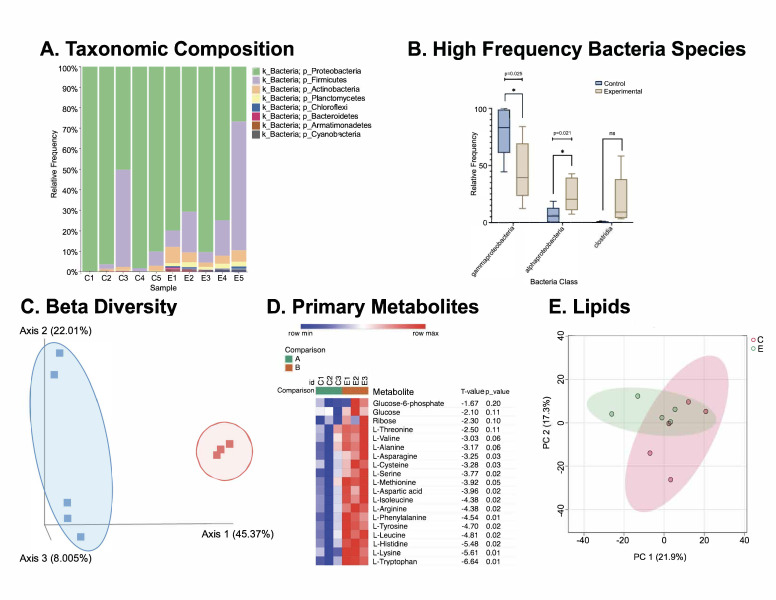



## Description

The human gut microbiome consists of a diverse collection of bacteria, viruses, archaea, eukarya (Botta et al., 2021). These microorganisms not only inhabit the gut, but they can provide the host with valuable nutrients needed to fulfill specific metabolic functions (Wu and Wang 2019). Specifically, the microbial community located within the gut has become an important area of research due to profound implications on disease modulation. The gut microbiome is dynamic and can be quickly altered by a number of factors, including age, geographical location, genetic variation, and diet (Adak and Khan 2019). With broad impacts on human health, it is essential to elucidate the impact of controlled diets on the gut microbiome composition.

Iron deficiency anemia, one of the most significant nutritional deficiencies worldwide, results from the insufficient consumption of iron. Under homeostatic conditions within the body, iron performs several important functions that are essential for life, such as transporting oxygen to tissues, performing electron transport in cells, and supporting enzymatic function within tissues of the body (Gupta 2014). As a result, a lack of iron can induce harmful results, including negative effects on growth and cognitive performance in infants, fatigue, weakness, and trouble concentrating in adults, and premature births in pregnant women (Piskin et al., 2022). To counteract the effects of insufficient iron levels, oral iron supplementation is the most common treatment. There are several ways to supplement iron, including micronutrient powders that can be added to foods before consumption. However, a common issue with iron supplementation is the low absorption that can occur within the small intestine, which creates an iron rich environment in which the microbiota exist (Jaeggi et al., 2015). Not only is iron needed by the body, but it is an important growth factor for many microorganisms such as pathogenic bacteria that have developed methods to competitively sequester iron from the body using siderophores (Caza and Kronstad 2013). Effects of this supplementation on the gut microbiome composition remain unknown, so it is important to conduct experimentation to better understand what occurs.

The aim of this study was to examine the impact of iron supplementation on the taxonomic composition and functional output of the gut microbiome using Zebrafish ( *Danio rerio* ) as a model species. Zebrafish are an ideal model organism as they have a fully sequenced genome and a large portion of their genome overlaps with that found in humans (Flores et al. 2019). To conduct these studies, two groups of zebrafish (same age and genotype) were separated and fed fixed amounts of iron. The first group represented the control group and received a standard amount of iron within their diet (309 ppm). The second group represented the experimental group and received a 19% increase in iron within their diet (366 ppm). The gut contents of both groups were then analyzed to evaluate differences in taxonomic composition and gut function.

We first focused on bacteria taxa that were present within the guts of the fish to determine if an increase in dietary iron significantly altered the relative frequency of particular taxa present within the gut microbiomes. Of the bacteria taxa, Proteobacteria were found at the highest relative frequency within the control group ( Figure 1A ). Several other classes were present, but were found at lower relative frequencies. Within the experimental fish, the three main classes of Gammaproteobacteria, Alphaproteobacteria, and Clostridia were found at the highest relative frequencies ( Figure 1A ). To analyze taxonomic differences between the control and experimental groups, the relative frequencies of these three classes were compared using a t-test ( Figure 1B ). Gammaproteobacteria frequencies significantly decreased within experimental specimens as compared to control specimens (p<0.025) while Alphaproteobacteria frequencies significantly increased (p<0.021). Both Gammaproteobacteria and Alphaproteobacteria belong to the larger phylum of Proteobacteria. Proteobacteria, the largest bacterial phylum, is made up of six classes of gram negative bacteria that are able to withstand a variety of oxic conditions. These bacteria are common within the gastrointestinal tract, and are able to decrease the redox potential within the gut by consuming oxygen thereby likely allowing for colonization by other strict anaerobic bacteria (Moon et al., 2018). Proteobacteria have also been implicated in several disorders including inflammatory bowel disease (Rizatti et al., 2017). There was no significant change in the frequency of clostridia between control and experimental groups. A Jaccard PCoA plot was generated to analyze similarities between individual control and experimental fish gut microbiome taxa ( Figure 1C ) and we found that members of the control group were more similar to each other than the experimental group. Our finding that samples from the normal iron group and high iron group formed 2 separate groups based on the PCoA supports the hypothesis that increased dietary iron consistently alters the taxonomic composition of the gut microbiome.

A replicate of the original experiment was performed with control and experimental fish receiving standard amounts of iron in their diet, and a 19% increase of iron in their diet respectively. Primary metabolite and lipid data extracted from the gut contents of these fish was then taken into further analysis.

Analyzing primary metabolites found within the gut helps to model byproducts created by present bacterial species. Bacteria within the gut aid in digestion of consumed material and in turn produce microbial-derived metabolites (Swer et al., 2022). As one of the main goals of the study was to search for any functional differences in the gut microbiome related to iron supplementation, primary metabolites were recorded. Several amino acids were found to be significantly increased within experimental specimens including L-Asparagine, L-Cysteine, L-Serine, L-Methionine, L-Aspartic Acid, L-Isoleucine, L-Arginine, L-Phenylalanine, L-Tyrosine, L-Leucine, L-Histidine, L-Lysine, and L-Tryptophan ( Figure 1D ). Similarly to primary metabolites, lipids found in the gut can also contribute to a greater understanding of any functional changes that might have occurred. However, after further comparison of lipid data between control and experimental groups, there was no significant difference found ( Figure 1E ).

Some limitations include not grouping fish by sex and using different individual fish for the microbial taxonomy and metabolomics analyses. Separating the experimental and control groups into different tanks could have introduced tank effects in addition to the effects of dietary iron on the observed results.

In summary, Gammaproteobacteria, Alphaproteobacteria, and Clostridia were found in the highest frequencies amongst experimental samples. A significant decrease of Gammaproteobacteria occurred, while a significant increase of Alphaproteobacteria occurred from control to experimental samples. Beta diversity measurement revealed greater diversity between control and experimental groups rather than within the groups. Several primary metabolite concentrations significantly increased within the experimental samples while there was no significant change in the lipids.

## Methods

*Zebrafish Care and Experimentation*

Zebrafish ( *n* =10) of the same age and genotype (AB wild type) were fed Ziegler Brothers Adult Zebrafish Food containing 309.00 ppm of dietary iron for two weeks. After two weeks, the fish were separated into the control and experimental groups and co-housed as those two groups. The control group continued to receive the initial feed while the experimental group received feed supplemented to 366 ppm of dietary iron (ferrous sulfate) to give an elevated iron level (a 19% increase in iron). After four weeks of these diets (6 weeks total), the fish were sacrificed, dissected distal to the gut bulb, and a 150 μL gut contents slurry was harvested in 1mL of 1X PBS for DNA extraction. A subsequent replicate trial of ten zebrafish was exposed to the same treatment (feeding regime and fish tanks), and gut contents were harvested after feeding trials concluded. The samples from these fish were used for metabolite and lipid extraction.

*Gut Content Analysis*

The suspended gut contents were sent to MRDNA ( https://www.mrdnalab.com ) for DNA extraction and 16s rRNA amplicon sequencing for identification of bacterial taxa within the V4 hypervariable region. Additionally, Dada2 (package 1.16.0) was utilized to infer exact amplicon sequence variants (ASVs) from high-throughput amplicon sequencing data. Sequenced DNA was analyzed computationally using QIIME2 (Quantitative Insights Into Microbial Ecology, version 2020.2.0) to translate raw sequence data into statistical results.

After another replicate zebrafish trial, gut contents were suspended and sent to Clemson University's Multi-User Analytical Lab for further analysis of primary metabolites and lipids. Three samples each from the experimental (higher iron) and the control group were of sufficient quality and quantity for analysis. Samples were analyzed with the Agilent 7250 Gas Chromatography/Quadrupole Time-of-Flight (GC-QTOF) and liquid chromatography mass spectrometry (LCMS) with a C30 LC column connected to a high resolution Orbitrap Fusion Tribrid mass spectrometer. Samples were further extracted with 600 uL methanol by homogenizing with ceramic beads in a CryoLys homogenizer. Water was added (450 uL) to achieve a sample composition of 50% methanol, and phase separated with 600 µL chloroform. Samples were inverted to mix, cooled on ice, and centrifuged 12,000 rpm at 15 ^o^ C for 5 min. Three-phase separation was formed with the methanol-water layer on top, tissue material interface in the middle, and chloroform layer at the bottom. For primary metabolomics analysis, 40 µL of the 50% methanol sample extract was aliquoted to glass insert, dried under nitrogen gas, and derivatized with MSTFA 1% TMCS for GC-QTOF analysis. Data was processed in MSDial, and compounds were tentatively identified using Kovats retention index GC-MS mass spectral library. For lipidomics analysis, 100 µL chloroform sample extract was diluted with 100 µL methanol in a glass insert and further analyzed on the Orbirap Fusion Tribrid mass spectrometer. Data was processed in LipidSearch for lipid identification. Three samples from the experimental (higher iron) and five from the control group were of sufficient quality and quantity for analysis.

*QIIME2 Analysis*

Both the taxonomic bar plot and Jaccard Principal Coordinates Analysis plots (PCoA) were generated using QIIME2. The taxonomic bar plot was created with bacteria class data from 16s rRNA was sequenced from zebrafish gut contents, and it was organized by relative frequency of the bacteria within each sample. The PCoA plot relied on the presence or absence of bacteria classes to measure the beta diversity between control and experimental groups.

*Box and Whisker Plot*

A box and whisker plot for bacteria class frequency among groups was created using GraphPad Prism version 10. To measure the statistical significance, a t-test calculator for two independent means was used ( https://www.socscistatistics.com/tests/studentttest/default2.aspx ). The threshold for significance was set to 0.05.

*Heatmap Generation*

Metabolic analysis data from Clemson’s Multi-User Analytical Lab were received, and a heatmap of the metabolomic levels was created using Morpheus matrix visualization and analysis software from the Broad Institute ( https://software.broadinstitute.org/morpheus ). T-tests were performed with this software to test for significant changes in metabolite concentrations.

*Principal Component Analysis*

Lipid analysis data were normalized by sum to adjust for systemic differences among samples. Normalized lipid data was used to generate a principal component analysis plot in order to examine differences in lipid concentrations between control and experimental groups.

## Reagents

**Table:** 

**Strain**	**Genotype**	**Available from**
AB wild type	*Danio rerio*	ZIRC
